# Complex behavioral plasticity is not reduced in spiderlings with miniature brains

**DOI:** 10.1371/journal.pone.0251919

**Published:** 2021-06-16

**Authors:** Rosannette Quesada-Hidalgo, William G. Eberhard, Gilbert Barrantes

**Affiliations:** 1 Escuela de Biología, Universidad de Costa Rica, San José, Costa Rica; 2 Smithsonian Tropical Research Institute, Panama, Panama; 3 Museum of Natural Science, Louisiana State University, Louisiana, United States of America; University of Vienna, AUSTRIA

## Abstract

The brains of smaller animals are smaller than those of their larger relatives, but it is not clear whether their adaptive behavioral flexibility is more limited. Previous interspecific comparisons found that aspects of web construction behavior of very small orb weaving spiders (0.005 mg) were no less precise than those of much larger related orb weavers (30 mg), but the behaviors tested were relatively simple. Here we perform a more sensitive intraspecific test involving the multiple behavioral adjustments of orb web designs made by *Leucauge argyra* to confinement in very small spaces. Web adjustments of spiderlings as small as ~0.1 mg were compared to previously published observations of ~80 mg conspecific adults. Spiderlings in constrained spaces made all of the complex adjustments made by adults in at least seven independent web design variables, and their adjustments were no less precise. Rough estimates based on previously published data on total brain volumes and the mean diameters of neuron cell bodies suggested that spiderlings and adult females of *Leucauge* may have similar numbers of neurons, due to spiderlings having smaller neurons and a greater percentage of body tissues dedicated to the brain. We speculate that this neural similarity may explain why *L*. *argyra* spiderlings showed no behavioral deficits compared with adults.

## Introduction

The sizes of animals vary greatly, with body weights spanning a range of about 10^14^ gm. Small animals generally have smaller brains, but smaller brains are often not simply miniaturized versions of larger brains [1]. They often have smaller and/or fewer neurons and synapses [summary in 2], and thus seem likely to be inferior in several respects [3]. For instance, reduced numbers of neurons may be less able to execute parallel processing and store memories [4,5]. These differences in brains raise the question of whether smaller animals have reduced behavioral abilities (the “behavioral deficit” hypothesis). Possible deficits could involve abilities to learn, or pre-programmed abilities to react flexibly to environmental variations. Alternatively, they may have evolved compensatory mechanisms and show no overall behavioral deficits.

The possibility of behavioral deficits in smaller animals has many important consequences for understanding basic topics in biology such as foraging ecology, sexual behavior, and the evolution of body size. However, precise and comparable quantification of different types of behavior, especially of complex behavior patterns that would seem more likely to be constrained by brain differences, is often difficult. Furthermore, correcting for possible phylogenetic inertia, and for differences in the difficulty of tasks and learning abilities in comparisons between species is also difficult. Studies of behavioral abilities of miniature arthropods that are sufficiently detailed to allow tests of the behavioral deficit hypothesis are very rare.

Surprisingly, the limited data suggest that smaller animals tend not to show behavioral deficits. For example, individuals of *Nephanes titan* (Ptiliidae) feather wing beetles, one of the smallest free-living insects, needed only two trials to learn to associate visual cues (white vs. grey filter paper) with food [6]. Nonetheless, their behavior was not compared with that of larger relatives. The parasitic wasp *Trichogramma evanescens* that weighs only about 0.05 to 0.09 mg, showed a “normal” range of behaviors that included flight, walking, courtship, deciding over the size and sex of their progeny, vision, olfaction, learning and long- and short-term memory formation [7], but there are no quantitative comparisons available of either the precision or elaborateness of their behavior with that of larger relatives. Previous studies of ants both supported [8,9] and contradicted [10] the behavioral deficit hypothesis, but in all cases depended on unsatisfactory estimates of the “complexity” of different behaviors [see discussion in 11].

Detailed studies of the web construction behavior of orb weaving spiders, which is expressed as soon as the spiderling emerges from the egg sac and shows substantial plasticity in response to environmental variations but little sign of learning effects [summary in 12], also gave no evidence of behavioral deficits in smaller individuals. When the webs of spiderlings and adults of three species whose weights varied by a factor of 10^4^ were compared, those of smaller spiders did not show greater imprecision, as measured by the consistency of the spacing between the sticky spiral loops [13]. When three orb web parameters thought likely to be affected by brain constraints were compared between spiderlings, older immatures and adults of two other species of orb weavers that differed in size, the spiderlings showed no evidence of being behaviorally more limited or more prone to make errors [14]. When the precision in webs built by spiderlings of different sizes and of adults of four species of orb weavers was compared by quantifying the scatter of correlations between different web variables, very small spiders showed one partial confirmation but failed to show three other trends predicted by the behavioral deficit hypothesis, and showed four additional trends that contradicted the behavioral deficit hypothesis; they also displayed additional flexibilities in web traits that were lacking in the larger spiders [11]. On the other hand, the results of a study of learning, rather than of presumably pre-programmed behavior, contrast with these findings. Small spiderlings of *Pholcus phalangioides* were apparently less able to retain memories of lost prey, as judged by the time spent searching when searches were postponed experimentally for periods of up to 16 min [15].

All of the studies just mentioned were limited to relatively simple and perhaps undemanding behavioral tasks [as noted by 11,13,14]. The present study attempts to remedy this problem by focusing on the suite of behaviors involved in extreme, coordinated flexibility in multiple, largely independent orb web traits, and that thus seems likely to be more neurally demanding. We compared strictly equivalent, complex behaviors that are minimally influenced by learning [12,13] among conspecific individuals in standardized conditions by studying different developmental stages of a single spider species, *Leucauge argyra* (Walckenaer 1842). We thus largely controlled for possible problems involving phylogenetic inertia, equivalence in tasks, and quantification of responses.

**Fig 1:**
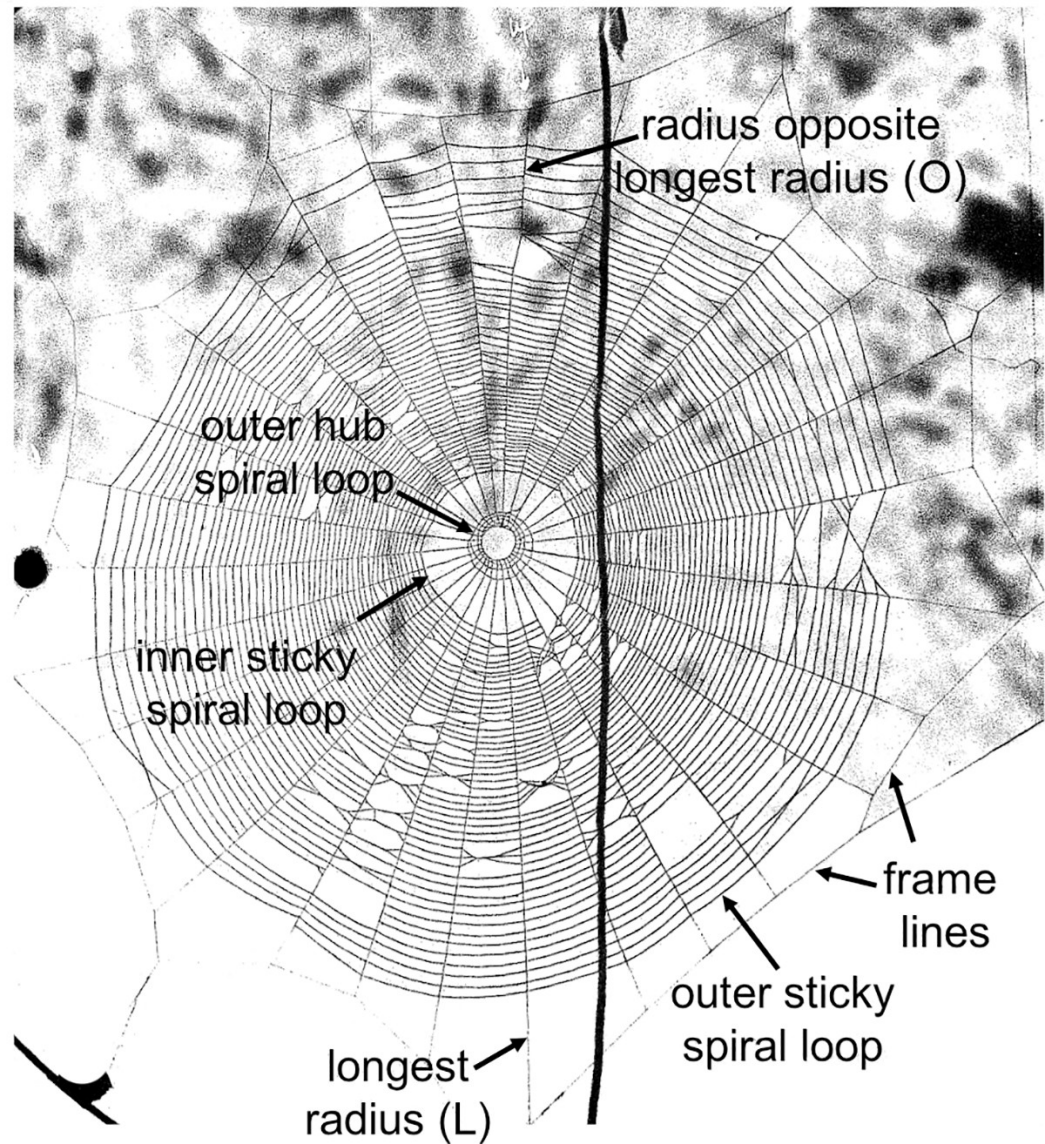
Orb web of a mature *Leucauge argyra* female built in the field with web measurements labeled.

Adult females of *L*. *argyra* normally construct standard orb webs in the field that are more or less horizontal and span approximately 80 to 100 cm across (Fig 1); but they built highly altered orb webs when they were confined in constrained spaces that were as small as only 7% of the mean span of webs in the field [16]. Spiders in these constrained spaces made many modifications that adapted their webs to these spaces, such as reduced spacing between sticky spiral loops, reduced numbers of frame lines, radii, and loops of sticky and hub spirals, and increased proportions of radii that were attached directly to the substrate. The present study tests whether spiderlings of this species are able to accomplish the same extreme behavioral adjustments to similarly constrained spaces, and with the same precision as conspecific adults [16]. Small spiderlings of *L*. *argyra* weigh close to 100 times less than adult females. This large size difference, the fact that both early instar spiderlings and adults perform the same behavior (orb construction) in nature, and the extreme behavioral plasticity of adults when they build orbs in constrained spaces, make *L*. *argyra* a useful model for comparative studies of possible behavioral deficits in small individuals. The behavioral deficit hypothesis predicts lack of correlation or lower slopes in correlations between web traits with increasingly constrained spaces.

## Methods

### Webs in the field

Orb webs built by small *L*. *argyra* spiderlings found in the field were photographed after coating them lightly with talcum powder. The spiderlings probably belonged to instars 2 (the first instar outside the egg sac) (R. Quesada personal observations) to 6, and were categorized based on their body size (see below). Second instar spiderlings disperse from the egg sac and build orb webs to capture prey [17]. Web photographs were taken in a plantation of African oil palm (*Elais guineensis* L.) near Parrita, Puntarenas, Costa Rica (09°30´N, 84°10´W, el. 10 m) from May to September 2011 and from February to June 2012. A black cardboard sheet was placed behind the web to improve contrast, and a calibrator was held next to the web to scale the photo. Spiderlings in the field built approximately horizontal webs that spanned attachment points 6 to 20 cm apart. Webs of spiderlings had a sparse tangle of non-sticky lines above the orb that was connected to the inner spirals of the hub; this tangle may provide support for the orb [18]. The size of the tangle and the number of threads that it contained were reduced in larger instars, and tangles were absent in the webs of adults [8]. Spiderlings from the photographed webs and conspecifics of similar sizes were collected for observations in captivity (Universidad de Costa Rica, San José province, el. 1200 m).

### Orb webs in constrained spaces

In the laboratory, we placed each spiderling in a vertically oriented cylindrical container with one of the following dimensions: (a) 24 mm diameter x 30 mm long, (b) 18 x 30 mm, (c) 9 x 15 mm and (d) 5 x 15 mm. One end of the 5 and 9 mm diameter cylinders was placed on a thin layer of white plasticine, and a glass coverslip was placed on the upper end to prevent the spider from escaping. The 18 and 24 mm diameter cylinders were covered at both ends with plastic wrapping material. Spiderlings were assigned to the containers randomly with respect to their sizes. All cylinders were lined with black paper in the upper third, to allow the spider to attach threads and to provide a dark background for photographs. The cylinders were kept in larger, 21 x 11 x 9 cm plastic containers with a piece of wet cotton to provide a humid environment. No spider was used more than once. Only two spiderlings built structures that resembled an orb web rather than simple accumulations of draglines in the 5 mm diameter containers, and neither was a complete orb, so only data from the 9 mm, 18 mm and 24 mm diameter cylinders were analyzed.

**Fig 2:**
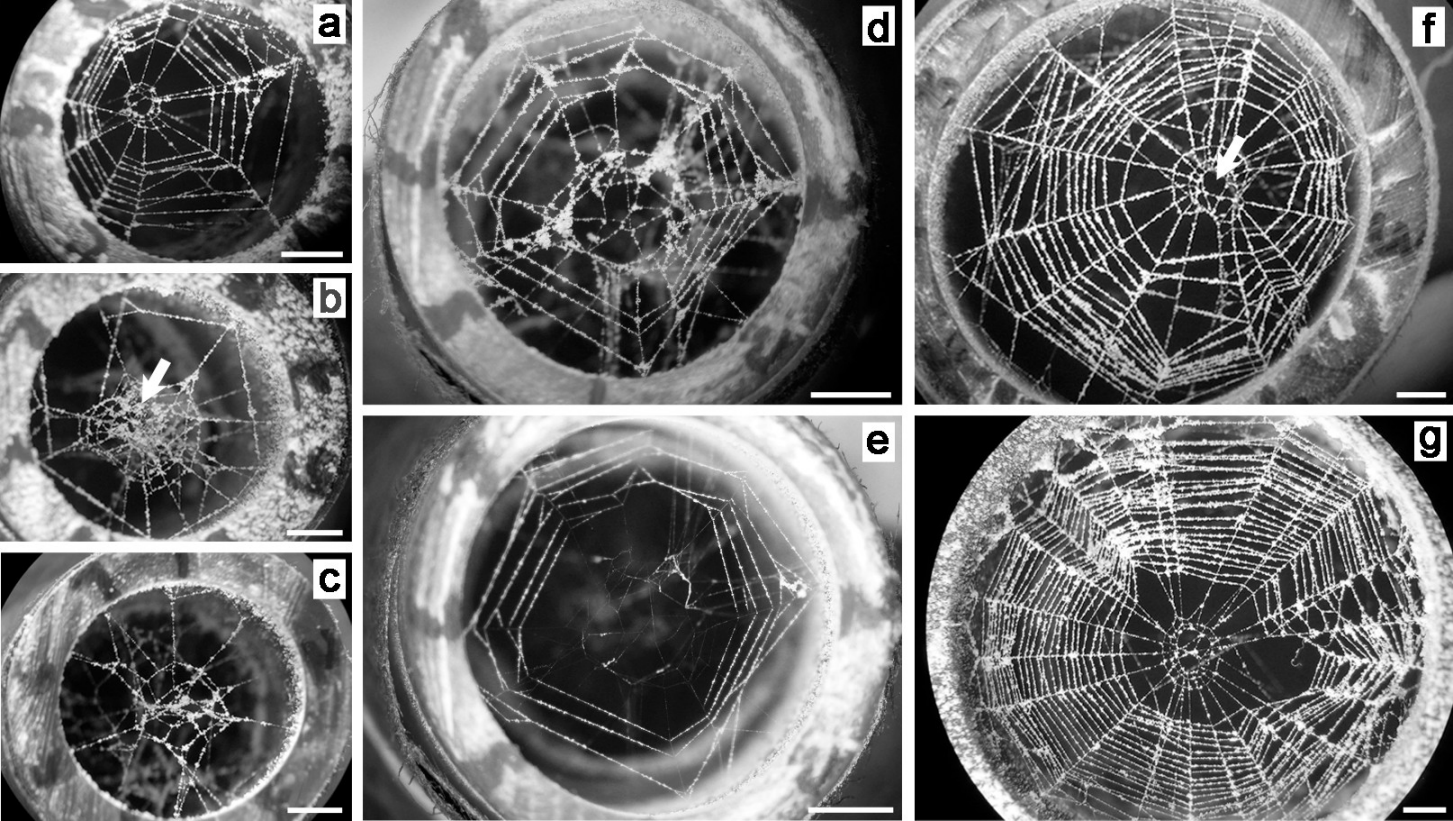
Orb webs built by *Leucage argyra* spiderlings in cylinders of different diameters in the laboratory. a-e) Orbs in 9 mm diameter cylinders: a) with many sticky spiral loops, b) with only one sticky spiral loop and an intact hub center (arrow indicates tufts of silk that accumulated during radius construction), c) with no sticky spiral, d) immediately after being coated with powder, e) after rapping the cylinder gently to displace the powder from the non-sticky lines; f) orb in a 18 mm diameter cylinder (white arrow indicates the hole where the center of the hub was removed); and g) orb in a 24 mm in diameter cylinder. All scale bars are 2 mm.

Webs built in the cylinders were photographed under a dissecting microscope after being coated with talcum powder; they were photographed again after the cylinder was jarred gently to cause the powder to fall from the non-sticky lines but remain on the sticky spiral loops (Fig 2D and 2E). Some spiders built their orbs on the first day after being introduced into their containers, while others built them up to 15 days later. Spiders that died within the first three days of being confined in a cylinder without building a web were excluded from the study. Those that died after the first three days without building an orb were counted as having failed to build an orb. All spiders were preserved in 70% ethanol for later body measurements.

### Categories of spiderling size

**Fig 3:**
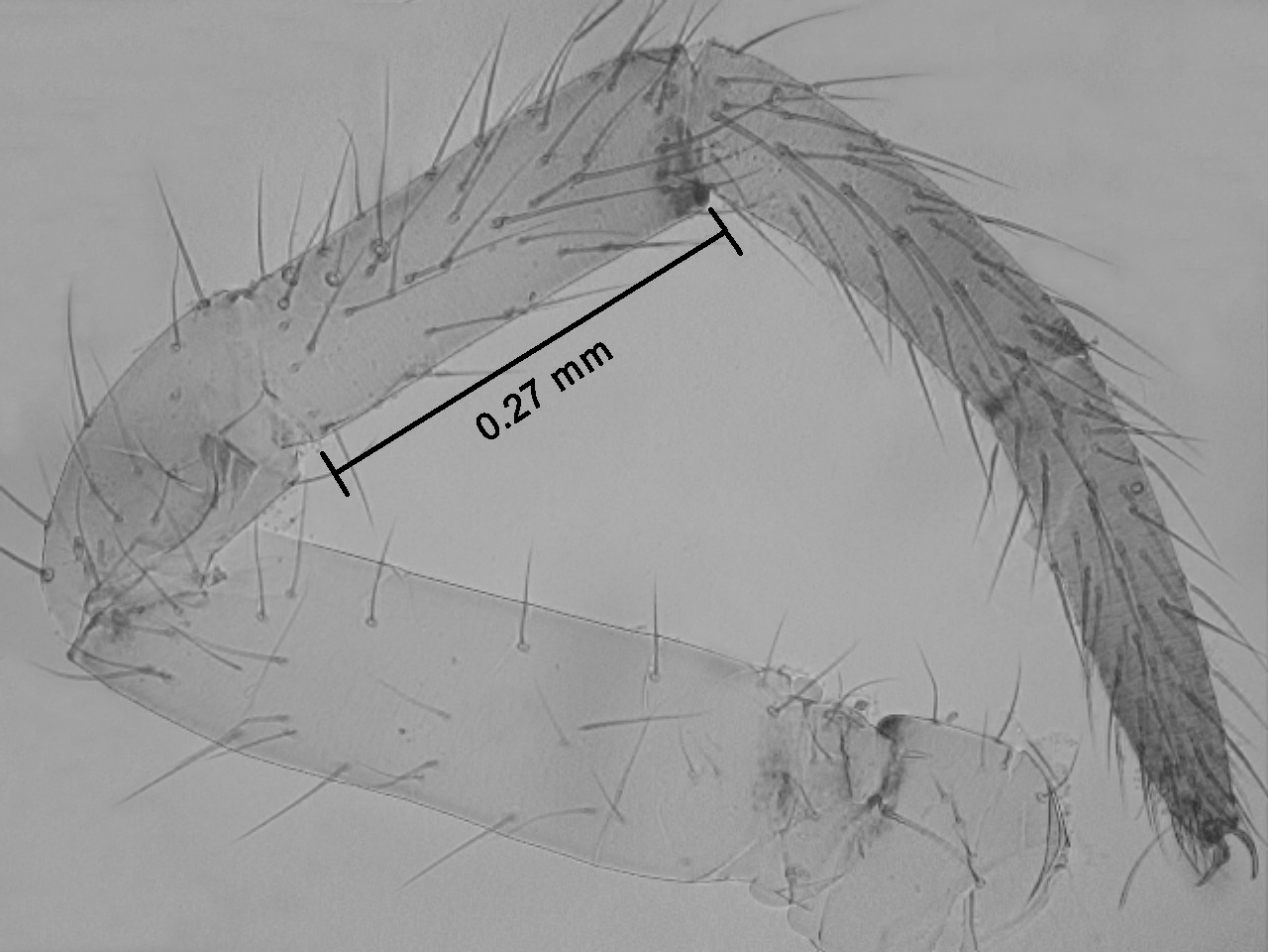
Right leg I of a *Leucauge argyra* spiderling on a slide. The length of the ventral margin of the tibia was measured as a proxy for body size.

**Fig 4:**
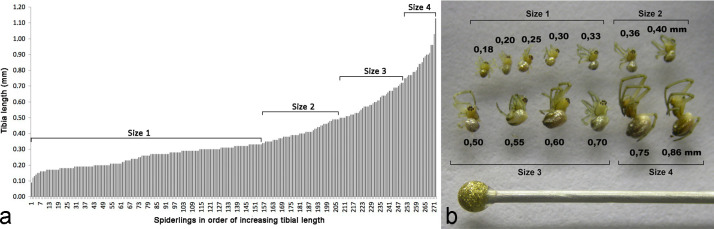
a. *Leucauge argyra* spiderlings in order of increasing tibial length showed a continuum in which no clear groups could be easily defined. The four body size categories are indicated. b. Examples of *L*. *argyra* spiderlings in the four body size groups, with their corresponding tibia lengths and the head of an insect pin for scale.

We measured the length of the ventral edge of tibia I as a proxy of body size in 271 spiderlings that built a web in the field or captivity, using photographs of the right leg oriented horizontally using a coverslip on a slide under a microscope (Fig 3) (in the few cases in which the right leg I could not be used we used the left one). We preferred this measure over measurements of cephalothorax length or width used in some other studies because it allowed greater ease of obtaining consistent, highly precise orientation. The distribution of tibial lengths was continuous, making it impossible to separate different spiderling instars confidently (Fig 4A). Therefore, individuals were placed in four arbitrary body size groups with the following tibia lengths (approximate weights of representative spiders are in parentheses): size 1: 0.12–0.33 mm (0.10–0.20 mg); size 2: 0.34–0.49 mm (0.20–0.30 mg); size 3: 0.50–0.72 mm (0.30–0.60 mg); and size 4: 0.73–1.03 mm (0.60–1mg) (Fig 4B). Size 1 spiderlings probably included second and third instar individuals, because there were two similar size subgroups. Size differences between the early instars of *L*. *argyra* are probably smaller than those in later instars, as occurs in other, distantly related spiders such as *Tengella radiata* (Zoropsidae) [19]. Adult female *L*. *argyra* had tibial lengths of approximately 3.4–4.1 mm and weighed approximately 80 mg. Therefore, the legs of the largest spiderlings in this study were only about 25–30% of the length of the adults and these spiderlings weighed about 80 times less than adults; the tibial lengths of the smallest spiderlings were only 3–4% of those of the adults and these spiderlings weighed approximately 500 times less than adults.

### Web variables

We used Image Tool [20] to measure the same web variables in field and lab photographs (Figs 1 and 2) that were used by Barrantes & Eberhard [16] to analyze adult *L*. *argyra* webs, in order to allow direct comparisons: the total area (area enclosed by the outer sticky spiral loop); the capture area (the total area minus the area enclosed by the inner sticky spiral loop); the free zone area (the area between the inner sticky spiral loop and the outer loop of the hub); the hub area (the area enclosed by the outer spiral loop of the hub); the number of radii (all radial lines ending in the hub and to which the outermost hub spiral was attached, whether or not they had sticky spirals attached to them; lines arising from the inner spirals of the hub going upward to a tangle or downward to the substrate were not counted as radii); the maximum number of loops in the hub spiral (counts were made above, below, and to the sides of the hub, and the largest value was used in the analyses); the web symmetry (length of the radius opposite to longest radius /length of the longest radius); the number of frame lines (peripheral lines with radii attached to them); the number of frame lines that had sticky spiral lines beyond them/the total number of frames; the number of radii attached directly to the substrate/the total number of radii; the mean number of radii attached to each frame; and the number of frames with only a single radius attached/the total number of frames. In addition, we counted the number of sticky spiral loops crossing the longest radius (L), and the radius opposite the longest radius (O), and we measured the spaces between these loops on radius L. We calculated the consistency of the sticky spiral spacing on radius L as (the mean of the values for space_n_)/(space_n-1_ + space_n+1_)/2) [as in 16]. In cases where these variables could not be measured confidently on the specified radius (e.g., lines had become tangled), measurements were performed on an adjacent radius. We also calculated the proportion of the capture area, free zone, and hub area relative to the total area of the web. Finally, we recorded whether the hub center was removed or intact (data not included in the previous study) by checking for the white specks (tufts of accumulated loose silk) at the hub’s center after radius construction in photos of webs with the powder removed (Fig 2B). Both the adults and spiderlings of *L*. *argyra* removed these accumulations of silk when they ingested the hub center immediately after finishing the sticky spiral, leaving an empty hole (Figs 1 and 2F), but specks remained when the hub center was left intact (Fig 2B and 2C). Data on hub removal are presented in the Supplementary Material (S1 Fig).

### Comparisons between webs built by spiderlings and adults

We constructed general linear mixed models with a Gaussian error distribution (GLMMs; library lmerTest, R language) to test the effect of the total area of the web, spider stage (adults vs nymphs), and the interaction of these two fixed (predictor) factors on 17 response variables of webs that were constructed in restricted spaces and in the field. Additionally, we included the site in which the spiders constructed their webs (containers of different diameters and the field), and the sizes of the spiders ordered in five categories as random factors in the models. This type of model is appropriate because we are not interested in knowing the effect of particular container sizes or of the spider sizes on the web design; rather we are interested in whether the changes on web design made to restricted spaces were similar between the two spider stages (adults vs nymphs) [21]. We categorized the size of spiders in order to include the data from adults. We feel confident that the size categories rescue a large part of the spider size variation when the tibia is used as a proxy of spider size (see the discussion of size categories above).

### Precision of the changes between spiderlings and adults

The precision with which spiderlings and adults modified their webs according to the space that was available was estimated by the variability of the residuals in regressions, on the assumption that more precise modifications would result in a smaller scatter of residuals. To compare the degrees of precision between spiderlings and adults, we extracted the residuals from each variable included in the GLMMs, and then compared the variability of these residuals between the two categories of spiders, using the Fligner test [22]. We preferred this test because other similar tests require normal distributions of residuals, and the distributions of the residuals in some variables deviated from normality. We compared all web variables, even those lacking a statistically significant effect of total area.

All variables were log_10_ transformed to approach the assumption of normality of residuals; areas of the different portions of the web were converted to square roots prior to their log_10_ transformation. We used R statistical software version 3.6.0 [23] for all statistical analyses. All means are followed by ± one standard deviation.

## Results

### Comparisons between webs of spiderlings and adults

**Fig 5:**
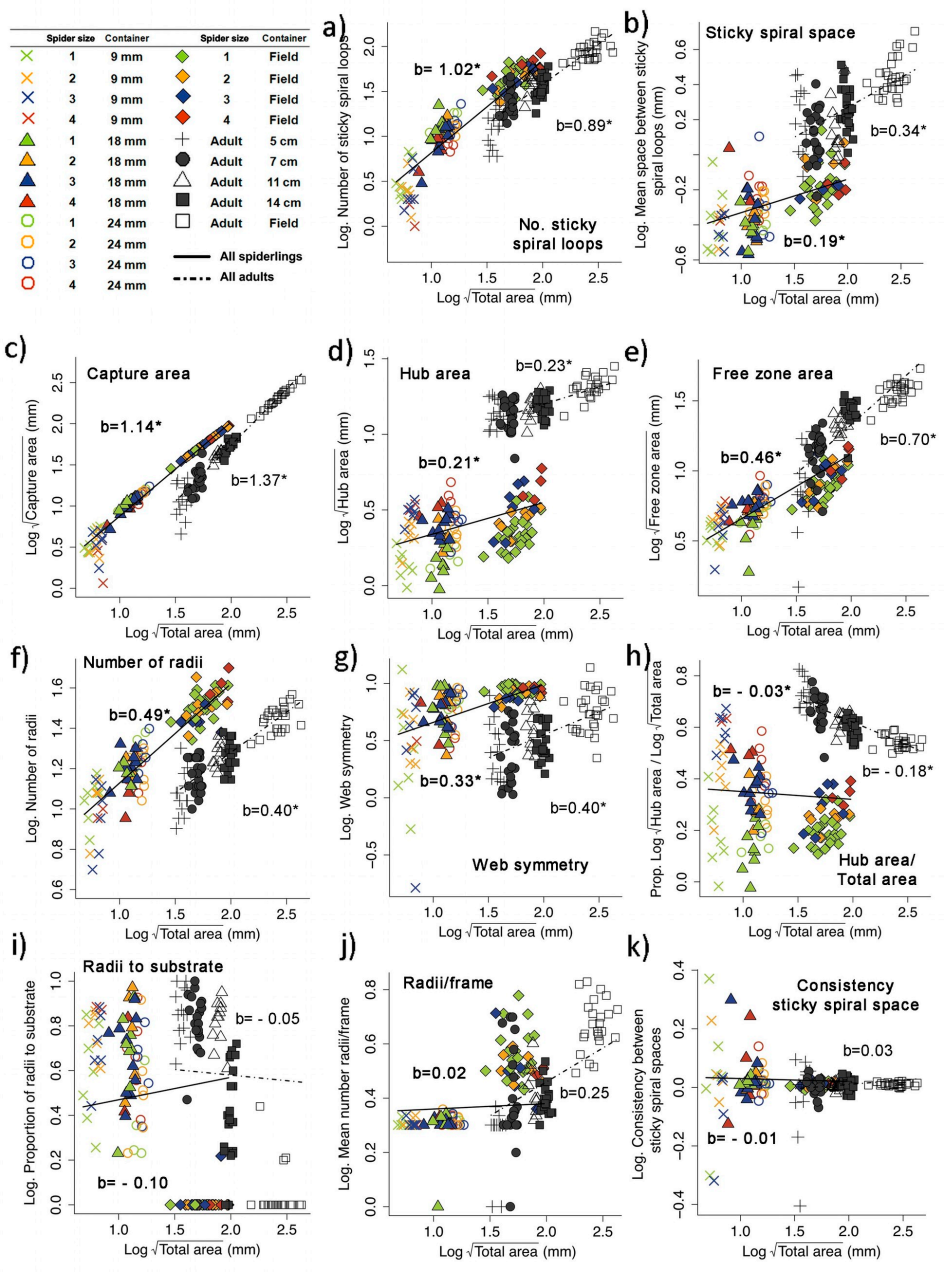
**Relationships between total web area and different variables measured in orb webs built in 9 mm, 18 mm and 24 mm diameter containers and in the field by *Leucauge argyra* spiderlings (colored symbols and solid lines) and in 5, 7, 11 and 14 cm diameter containers and in the field by conspecific adult females (black and white symbols and dotted lines).** Data on adults are from Barrantes & Eberhard [16]. The slope value (b) for all spiderling webs is in bold, and that for all adult webs in regular font. An asterisk indicates that the slope is statistically significant.

Representative orbs in each size of container are shown in Fig 2. Including the size of the container in which the spiders constructed their webs and the sizes of the spiders as random factors in the GLMM analyses allowed us to compare the effect of the fixed factors on the web design of adults and spiderlings. The interaction between slopes (“stage*total area” in Table 1), included as a fixed factor in the models, had a significant effect on only one variable (capture area; Table 1; Fig 5C). For this variable, the slope of spiderlings was significantly lower than in adults (Table 1): spiderling webs had relatively smaller capture areas. There were marginally significant interactions for three additional variables (number of radii, free zone/total area, hub area/total area); but even if these are considered as significant, which is arguable, the general pattern remains, because slopes for nymphs of number of radii and hub area/total area were higher rather than lower, and only the free zone area/total area was lower.

**Table 1:** Effect of total area of the web, spider stage (nymphs vs adults), and interaction (stage*total area) on log transformed web variables (as defined in Methods), using general linear mixed models, with spider size category and size of the container where *Leucauge argyra* spiderlings and adults constructed their webs as random factors.

Response variable	Coefficient	SE	df	t-value	p
**Capture area**					
Intercept	-1.00	0.16	11	-6.40	< 0.0001
total area	1.37	0.08	11	17.37	< 0.0001*
nymph-adult	0.74	0.18	12	4.17	0.00129*
stage*total area	-0.23	0.10	13	-2.32	0.03760*
**Free zone area**					
Intercept	-0.07	0.21	11	-0.32	0.755
total area	0.70	0.10	11	6.67	< 0.0001*
nymph-adult	0.27	0.24	12	1.13	0.281
stage*total area	-0.24	0.13	12	-1.77	0.101
**Hub area**					
Intercept	0.74	0.17	6	4.39	0.00551
total area	0.23	0.05	3	4.53	0.01658*
nymph-adult	-0.61	0.19	6	-3.23	0.01954*
stage*total area	-0.02	0.07	3	-0.24	0.82078
**Number of radii**					
Intercept	0.48	0.06	4	8.31	0.00173
total area	0.40	0.03	4	13.99	0.00044*
nymph-adult	0.16	0.06	4	2.44	0.08371
stage*total area	0.09	0.04	3	2.55	0.09319
**No. of hub loops**					
Intercept	0.44	0.09	5	4.69	0.0052
total area	0.03	0.05	5	0.64	0.5531
nymph-adult	-0.06	0.11	5	-0.59	0.5810
stage*total area	0.07	0.06	5	1.13	0.3102
**Web symmetry**					
Intercept	-0.25	0.23	6	-1.08	0.32100
total area	0.40	0.12	6	3.37	0.01641*
nymph-adult	0.58	0.27	6	2.16	0.07492
stage*total area	-0.07	0.16	6	-0.42	0.69234
**No. of frames**					
Intercept	-0.04	0.28	8	-0.16	0.8766
total area	0.44	0.14	7	3.11	0.0162*
nymph-adult	0.36	0.32	8	1.12	0.2975
stage*total area	0.01	0.18	7	0.04	0.9685
**Radii attached to substrate/total radii**					
Intercept	0.68	0.46	62	1.48	0.144
total area	-0.05	0.22	113	-0.23	0.817
nymph-adult	-0.31	0.53	45	-0.58	0.563
stage*total area	0.15	0.28	132	0.54	0.594
**Mean radii/frame**					
Intercept	-0.04	0.22	8	-0.208	0.8400
total area	0.25	0.11	9	2.25	0.0515
nymph-adult	0.38	0.24	9	1.56	0.1524
stage*total area	-0.23	0.14	11	-1.63	0.1301
**Frames w. single radius/total frames**					
Intercept	1.48	0.22	5	6.57	0.00185
total area	-0.38	0.11	4	-3.37	0.02549*
nymph-adult	-0.23	0.26	4	-0.91	0.40965
stage*total area	0.09	0.15	4	0.61	0.57867
**Capture area/total area**					
Intercept	0.39	0.18	22	2.19	0.0393
total area	0.23	0.09	22	2.61	0.0158*
nymph-adult	0.32	0.20	22	1.63	0.1174
stage*total area	-0.09	0.11	27	-0.80	0.4310
**Free zone/total area**					
Intercept	0.57	0.14	9	4.17	0.00266
total area	0.04	0.07	7	0.67	0.52506
nymph-adult	0.25	0.16	9	1.59	0.14568
stage*total area	-0.19	0.09	9	-2.13	0.06358
**Hub area/total area**					
Intercept	0.98	0.16	8	6.00	0.000325
total area	-0.18	0.06	7	-3.14	0.017718*
nymph-adult	-0.60	0.18	8	-3.25	0.011182*
stage*total area	0.15	0.08	8	1.96	0.087775
**No. sticky spiral loops L+O**					
Intercept	-0.20	0.28	18	-0.71	0.485
total area	0.89	0.14	17	6.33	< 0.0001*
nymph-adult	0.00	0.32	19	0.00	0.999
stage*total area	0.13	0.18	21	0.76	0.455
**Mean sticky spiral space L**					
Intercept	-0.41	0.17	7	-2.35	0.05307
total area	0.34	0.09	6	3.80	0.00836*
nymph-adult	-0.11	0.20	7	-0.54	0.60829
stage*total area	-0.15	0.12	7	-1.29	0.23888
**Consistency L**					
Intercept	-0.05	0.04	42	-1.12	0.2648
total area	0.03	0.02	42	1.20	0.2332
nymph-adult	0.09	0.05	42	1.84	0.0678
stage*total area	-0.04	0.03	42	-1.58	0.1160
**No. frames w. spiral lines beyond/total frames**					
Intercept	0.83	0.58	15	1.43	0.174
total area	-0.17	0.30	15	-0.58	0.568
nymph-adult	-0.45	0.66	16	-0.69	0.502
stage*total area	0.10	0.38	19	0.27	0.789

Data on adults are from Barrantes & Eberhard [16]. L = Longest radius; L+O = Longest radius plus radius opposite the longest radius; asterisks mark significant relations.

In contrast, the effect of total area as a fixed factor was significant but similar for spiderlings and adults in nine variables. The values of nine of the 17 variables, including the capture area whose interaction between stage and total area was significant, increased significantly with total area of the web (capture area, free zone, hub area, number of radii, web symmetry, number of frames, capture area/total area, number of sticky spiral loops on L+O, and sticky spiral space L; Fig 5), and they decreased with total area in two additional variables (frame with single radius/total frames, and hub area/total area; Fig 5). The variation in six additional variables (number of hub loops, number of radii attached to substrate/total radii, mean radii/frame, free zone/total area, consistency L, and number of frames w. spiral lines beyond/total frames) did not correlate with changes in total area in either spiderlings or adults (Table 1; Fig 5). Overall, the adjustments made by spiderlings and adults were thus similar.

### The precision of changes

The variability of the residuals was similar in spiderling and adult webs in 10 of the 17 variables (Table 2). The degree of the precision of modifications in five other variables, capture area, number of hub loops, web symmetry, the mean radii per frame, the spaces between sticky spiral loops, and the proportion of frames with a single radius/total frames, was larger (smaller variability of residuals) in spiderlings than in adult spiders (Table 2). Spiderlings were less precise in adjusting only two variables, the proportions of the web area dedicated to the free zone and to the hub (Table 2).

**Table 2:** Statistical comparison of the variability of residuals between spiderlings and adults that were obtained from the general linear mixed models, using the Fligner test.

Web variable	Fligner test	p	output
Capture area	5.68	0.0171	adults > spiderlings
Free zone	0.68	0.4094	adults = spiderlings
Hub área	0.97	0.3243	adults = spiderlings
Number of radii	0.61	0.4357	adults = spiderlings
No. of hub loops	5.69	0.01708	adults > spiderlings
Web symmetry	7.54	0.00601	adults > spiderlings
No. of frames	0.15	0.6957	adults = spiderlings
Radii attached to substrate/total radii	0.74	0.3902	adults = spiderlings
Mean radii/frame	32.85	< 0.0001	adults > spiderlings
Frame w. single radius/total frames	40.13	< 0.0001	adults > spiderlings
Capture area/total area	1.04	0.3086	adults = spiderlings
Free zone/total area	3.84	0.05016	spiderlings > adults
Hub area/total area	26.67	< 0.0001	spiderlings > adults
No. sticky spiral loops L+O	0.03	0.8448	adults = spiderlings
Sticky spiral space L	2.04	0.1532	adults = spiderlings
Consistency L	0.17	0.6793	adults = spiderlings
No. frames w. spiral lines beyond/total frames	0.03	0.8609	adults = spiderlings

Data on adults from Barrantes & Eberhard [16].

## Discussion

### Lack of behavioral deficits in spiderlings

Our results demonstrate that *L*. *argyra* spiderlings made most of the same adjustments to constrained spaces as conspecific adults (16 of 17 variables). The similarities included not only changes in the absolute values of web variables, but also changes in the proportions of different parts of their orbs. Adjustments to constrained spaces in both spiderlings and adults involved reduced numbers of frames, radii and sticky spiral loops, less space between sticky spirals loops, less capture area/total area, a greater proportion of frames with a single radius, and a greater proportion of the hub to total area (Table 1).

In addition, the adjustments by spiderlings were no less precise than those of adults, judging by the magnitudes of the residuals in regression analyses on web area (Table 2). The spiderlings’ residuals were even smaller than those of adults in five of the seven variables for which dispersion of residuals differed between spiderlings and adults (Table 2). In sum, spiderlings were not less behaviorally plastic by these measures. The adjustments documented here in spiderling webs, just as those documented previously in adult webs [16], represent continuations in trends present in field webs (S1 Table in S1 Appendix); thus the flexibility that we documented here was presumably due to pre-programmed responses to smaller spaces that likely resulted from natural selection in the field.

It should be noted that the lower limit in the space in which web construction behavior was elicited in spiderlings may be slightly larger (in relative terms) than that in adults. Based on the diameters of the spaces used in nature, spiderlings in captivity built in spaces with diameters 5–15% of those of field webs (S2 Fig), but not in spaces that were approximately 3–8% of field web diameters; adults built in spaces whose diameters were approximately 7% of the minimum web diameters in the field [16]. A difference in the threshold at which different spiders will build is not necessarily equivalent, however, to a difference in their abilities to make adjustments in web designs.

### Independence of different responses to constrained spaces

It is likely that some of the 17 variables we measured are inter-related, so probably not all the changes represent independent adjustments. In addition, changes in some earlier orb web elements may alter later stages of construction during the sequence of operations throughout orb construction (first the primary radii and frame lines, then the secondary frames, radii and hub, followed by the temporal and sticky spirals and finally hub removal) [12]. For instance, the number of frame lines, the size of the capture zone, the size of free zone and the size of the hub may all depend on the total web area and might not be independent. It is thus necessary to correct for possible inter-dependence to avoid overestimating the number of responses. Barrantes & Eberhard [16] discussed the question of independence of these variables. They concluded, based on three criteria (decisions are separated in time; decisions are influenced by different cues; and physical constraints do not impose similar decisions) and also on data from other studies, that the responses of adult *L*. *argyra* to constrained spaces involved (conservatively) at least seven biologically independent web variables. They noted that several lines of evidence indicate that orb web construction consists of semi-independent modules [summary in 12], and that not all web variables depend strictly on previous decisions made by the spider during construction. For example, even though the capture area in the present study was necessarily limited by the space available to build the web, the spider could nevertheless decide whether to leave larger or smaller spaces between the sticky spiral loops and whether to extend the sticky spiral closer to the hub (and thus reduce the free zone area). The implication is that the similarity in the adjustments made by spiderlings to constrained spaces that are documented in this study probably also involved at least seven independent types of decisions. The general conclusion is that the similarities between spiderling and adult responses documented here are substantially more complex than the similarities demonstrated between tiny and larger individuals in previous studies of orb construction [11,13,14], because they involved more independent behavioral traits.

Another point requiring clarification is that some variables in the present study are, in some senses, “biologically unrealistic”. Take, for instance, the variable “number of sticky loops”. It is very unlikely that spiders counted the number of loops of sticky spiral. This problem, however, does not necessarily lead to an overestimate of the number of independent variables. Changes in the number of loops in a web presumably resulted from several decisions, including how close to the end of the radius to attach the outermost loop of sticky spiral, how far apart to space subsequent loops from each other, and when to terminate sticky spiral construction.

### The precision of changes

The spiderlings’ ability to perform the same adjustments to constrained spaces with precision that was similar to that of adults echoes the results of other studies that showed that smaller spiders were no less precise in other aspects of orb web construction [11,13,14]. The changes in adjustments of web variables documented in the present study involved many different aspects of web construction. This study thus provides the most comprehensive data demonstrating similar behavioral abilities in small as compared with larger related animals. Moreover, most of the web changes made by spiderlings represented extensions of adjustments that spiderlings and adults make to less restrictive space limitations in the field [16; S1 Table in S1 Appendix]. This implies that the cues and the responses to these cues that were used by spiderlings to adjust their web designs to small spaces were probably the same as those used by adults. Nonetheless, our study also has limitations. We did not consider, for instance, the possible effects of size on the precision of regulating the amounts of adhesive or the diameters of lines, or the speed at which spiders worked, or the amount of exploration performed prior to construction. As far as the data go, they do not show the differences predicted by the behavioral deficit hypothesis. In sum, the central conclusion in this study is that the small sizes of spiderlings are not associated with behavioral deficiencies in flexibility in orb construction.

### Brains of spiderlings and adults

Lurking unmentioned in this discussion is the question of differences between spiderling and adult brain size and anatomy. While there are no data for *L*. *agyra*, extensive data exist for brain size and anatomy (“brain” volume is defined here as volume of the sum of the supra-esophageal plus the sub-esophageal ganglia) of the slightly smaller congeneric species *Leucauge mariana* [24]. In this species the estimated total volume of the brain of an instar 2 spiderling (that weighed about 0.1 mg, and would be in size category 1 of the present study) was 10.5 x 10^6^ cubic microns: 6.15 in the neuropil (the region of the brain containing axons and dendrites) and 4.35 in the cortex (the region surrounding the neuropil that contains the neuron cell bodies). The estimated brain volume for an adult female (weighing about 60 mg) was 240.9 x 10^6^ cubic microns (152.6 in the neuropil, 88.3 in the cortex) [24]. The percentage of the total brain volume that was cortex was nearly identical (36% vs. 37%) in spiderlings and adults, but the absolute volume of the spiderling’s brain was only approximately 4% of that of the adult. Rough calculations, using the data from the Quesada et al. [24] study, suggest that the brains of *L*. *mariana* spiderlings and adults may nevertheless contain roughly similar numbers of neurons. The mean diameter of an individual neuron cell body was 4.07 microns in the spiderling, and 10.72 in the adult female. Using the formula (4/3)πr^3^ for the volume of a sphere, which approximates the form of a neuron cell body (without its various processes, many of which are located in the neuropil), the estimated mean volumes of individual neuron cell bodies in the spiderling and in the adult would be 35.3 and 645 cubic microns respectively. As the estimated volume of the brain cortex was 4.35x10^6^ cubic microns in the spiderling, and 88.3x10^6^ cubic microns in the adult, the respective approximate estimated numbers of neurons in *L*. *mariana* are 123,000 in spiderlings and 137,000 in adults. Spiderlings and adults were also similar with respect to the fraction of the brain volume represented in the supra-esophageal ganglion (where analytical processes are thought to be more common): 36% of the total brain volume was in the supra-esophageal ganglion in the spiderling, and 33% in the adult.

These estimates are only approximations, and both neuron shapes and sizes likely differ in different parts of the brain. But if these differences are similar in spiderlings and adults, they may not seriously affect the estimates of neuron numbers. As far as they go, the data suggest that the brains of the spiderling and the adult have roughly similar numbers of neurons. This similarity in spiderling and adult *L*. *mariana* neuron numbers is due to the spiderlings having smaller neurons and also to their greater relative investment in brain tissue; the spiderling’s brain occupied about 48% of its cephalothorax volume, while that of the adult occupied only about 14% [24]. The relatively uniform nature of brain size allometries in spiders and other animals, especially among the members of a given taxonomic group [2,23,24] makes it very likely that the relation between the numbers of neurons in adults and spiderlings of *L*. *argyra* is similar to that in *L*. *mariana*. Calculations using data for an orb-weaver in a different family, *Anapisona simoni* (Anapidae) [24], also give similar estimated numbers of neurons in spiderlings and adult females (159,000 and 145,000 respectively).

We hypothesize that the comparatively similar estimated numbers of neurons in spiderlings and adults may explain the similarity in the complexity and precision of the behavioral adjustments that we documented here. This suggestion is only preliminary, however, as nothing is known regarding how (or if) brain volume and neuron numbers affect the flexibility of orb construction behavior. In addition, the possibility of behavioral deficits in *L*. *argyra* spiderlings with respect to learned rather than unlearned behavior [15] remains unstudied.

Generalizing to other species is also risky. Even if it is true that *L*. *argyra* spiderlings have avoided possible behavioral deficits associated with miniaturization by having smaller neurons and relatively larger investments in nerve tissue, this does not necessarily imply that these same tactics have been used in other animal groups to compensate for small size. It is not even well-established whether smaller individuals in other groups do or do not suffer behavioral deficits [25]. Much additional work, especially with respect to behavior, needs to be done.

## Supporting information

S1 Fig Frequency with which the center of the hub in orbs of *Leucauge argyra* spiderlings was removed as a function of a) the total web area and b) spiderling size, in orb webs built in 9 mm, 18 mm and 24 mm diameter cylinders and in the field. The red lines represent the predicted probability of removing the hub calculated by a logistic regression. Symbols above the line indicate webs whose hub centers were removed; those below the line had hub centers that were left intact.
(TIF)

S2 Fig Percentage of *Leucauge argyra* spiderlings that built an orb web in 9 mm, 18 mm and 24 mm diameter cylinders (sample sizes are given above each bar).
(TIF)

S1 Appendix Supplementary text, figures, and table. S1 Table. Web variables measured in orb webs built by *Leucauge argyra* spiderlings and adults in constrained spaces in the laboratory and the field, classified according to whether the adjustments made in the laboratory and in the field represented continuations of adjustments to smaller spaces made in the field. Data on adults from Barrantes & Eberhard [16]. The criterion used to define a continuation was lack of a significant difference between the slope in “allwebs” (webs built both in the laboratory and the field) by a given spider stage category (spiderling or adult) compared with the slope of only field webs of that category (indicated by “✓”). Statistically significant differences between all webs and field webs, implying that adjustments in the laboratory were not continuations of adjustments made in the field, are indicted by “X”.
(DOCX)
