# Supplementary figures and images for: Complex behavioral plasticity is not reduced in spiderlings with miniature brains
Source: PLoS One. 2021 Jun 16;16(6):e0251919. doi: 10.1371/journal.pone.0251919 (PMC8208555; doi:10.1371/journal.pone.0251919)

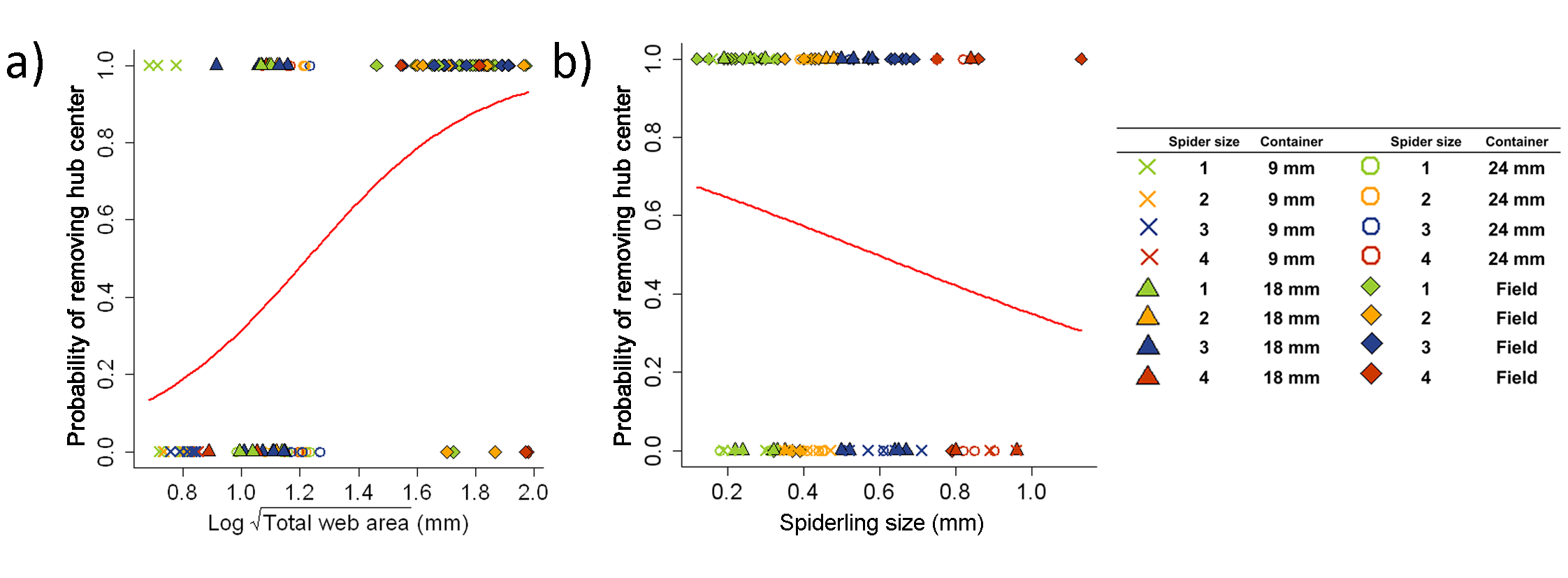

Supplement: S1 Fig — Frequency with which the center of the hub in orbs of Leucauge argyra spiderlings was removed as a function of a) the total web area and b) spiderling size, in orb webs built in 9 mm, 18 mm and 24 mm diameter cylinders and in the field. The red lines represent the predicted probability of removing the hub calculated by a logistic regression. Symbols above the line indicate webs whose hub centers were removed; those below the line had hub centers that were left intact. (TIF) [file pone.0251919.s001.tif]

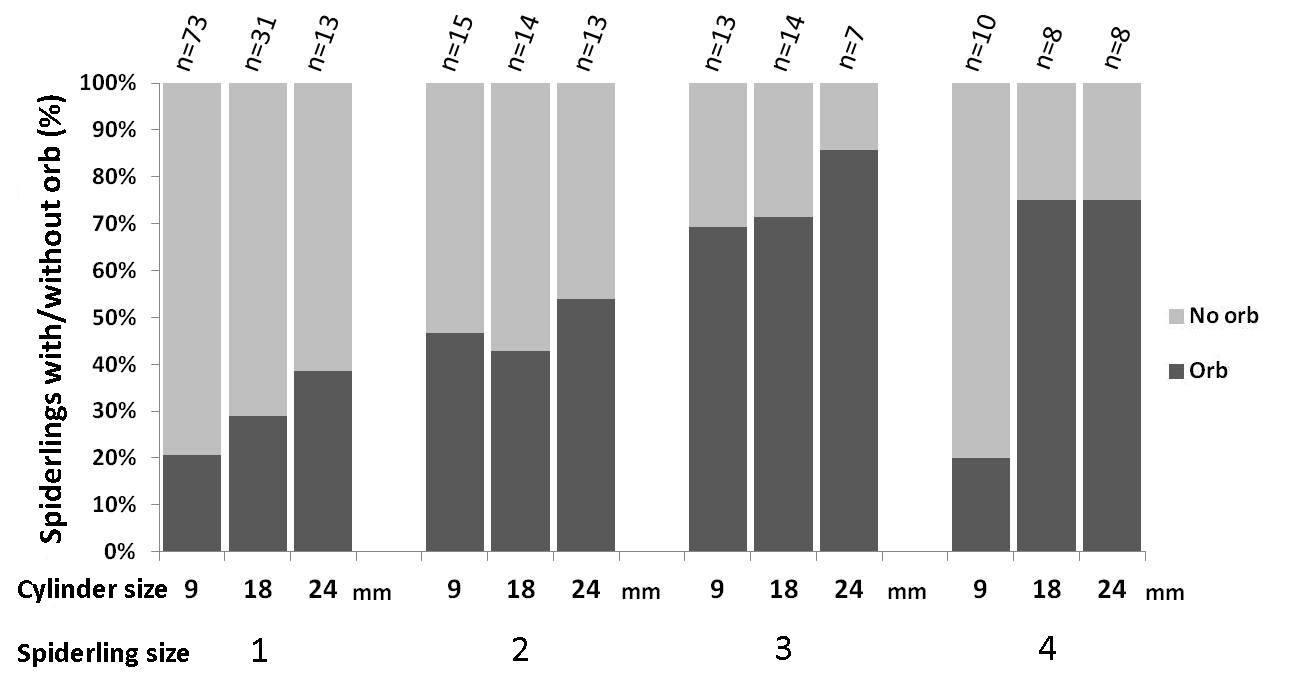

Supplement: S2 Fig — Percentage of Leucauge argyra spiderlings that built an orb web in 9 mm, 18 mm and 24 mm diameter cylinders (sample sizes are given above each bar). (TIF) [file pone.0251919.s002.tif]
