# Supplementary material for: Complex behavioral plasticity is not reduced in spiderlings with miniature brains
Source: PLoS One. 2021 Jun 16;16(6):e0251919. doi: 10.1371/journal.pone.0251919 (PMC8208555; doi:10.1371/journal.pone.0251919)
Supplement: S1 Appendix — S1 Table. Web variables measured in orb webs built by Leucauge argyra spiderlings and adults in constrained spaces in the laboratory and the field, classified according to whether the adjustments made in the laboratory and in the field represented continuations of adjustments to smaller spaces made in the field. Data on adults from Barrantes & Eberhard [16]. The criterion used to define a continuation was lack of a significant difference between the slope in “allwebs” (webs built both in the laboratory and the field) by a given spider stage category (spiderling or adult) compared with the slope of only field webs of that category (indicated by “✓”). Statistically significant differences between all webs and field webs, implying that adjustments in the laboratory were not continuations of adjustments made in the field, are indicted by “X”. (DOCX) [file pone.0251919.s003.docx]

**Supplementary Information**

**for Complex behavioral plasticity is not reduced in spiderlings with miniature brains**

**Hub removal in spiderling webs**

We recorded whether the hub center was removed or intact in webs built in constrained spaces and in the field by *L. argyra* nymphs, by checking for the white specks (tufts of accumulated loose silk) at the hub’s center after radius construction in photos of webs with the powder removed. Both the adults and spiderlings of *L. argyra* removed these accumulations of silk when they ingested the hub center immediately after finishing the sticky spiral, leaving an empty hole (Fig 2d), but specks remained when the hub center was left intact (Fig 2f). To test which variables were related to hub removal, we performed logistic regressions in which the dependent variable was whether the hub center was removed or intact, and the independent variables were: 1) total web area; 2) spiderling size, and 3) both total web area and spiderling size. Akaike information criterion (AIC) was then used to choose which regression better explained the probability of hub removal. The regression with the lowest AIC value was taken to be the most plausible model (regressions that differed from the best model by less than two AIC units were considered equally plausible). When two or more regressions were equally plausible, we chose the regression with the fewest predictors.

The probability of removing the center of the hub was significantly lower in spiderling webs with smaller areas than in webs with larger areas (x^2^=36.89, df=1, p< 0.0001, S1.a Fig). There was no significant difference in the probability of removing the center of the hub between the different size classes of spiderlings (x^2^=3.03, df=1, p=0.086, S1.b Fig). Similarly, the model of hub removal that used (1) only total web area as an independent variable was better (AIC=119.90) than the models that included (2) both the total web area and the size of the spider (AIC=120.33) and (3) only the spiderling size (AIC=153.75). No equivalent data on the frequencies of hub center removal are available for adults, so no comparisons were made. Nevertheless, intact hub centers were also observed in some adult webs in constrained spaces in captivity; they are rare (if they exist) in field webs (G. Barrantes & W. Eberhard, pers. comm.).


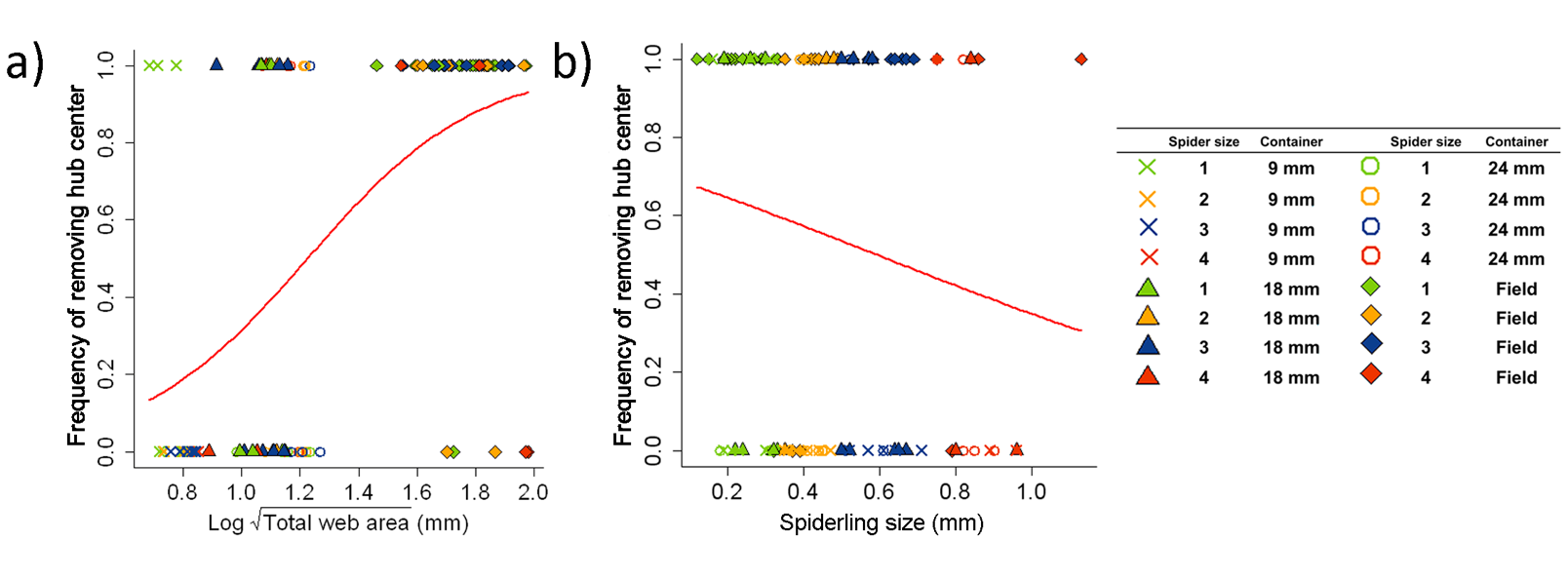


**S1 Figure.** Frequency with which the center of the hub in orbs of *Leucauge argyra* spiderlings was removed as a function of a) the total web area and b) spiderling size, in orb webs built in 9 mm, 18 mm and 24 mm diameter cylinders and in the field. The red lines represent the predicted probability of removing the hub calculated by a logistic regression. Symbols above the line indicate webs whose hub centers were removed; those below the line had hub centers that were left intact.

**Frequency of web construction in constrained spaces**

The frequency with which spiderlings built orb webs was higher in larger cylinders when spiderlings of all sizes were combined. Smaller spiderlings generally built proportionally fewer webs than larger ones in all three containers, except for the size 4 spiderlings, whose building rate slightly was lower than that of size 3 spiderlings. Based on a General Linear Model with a binomial distribution of errors, both spiderling size (t = 4.29, p < 0.0001) and available space (t = 2.61, p = 0.0098) in the container (but not their interaction) affected the probability that spiderlings would build a web (S2 Fig).

**
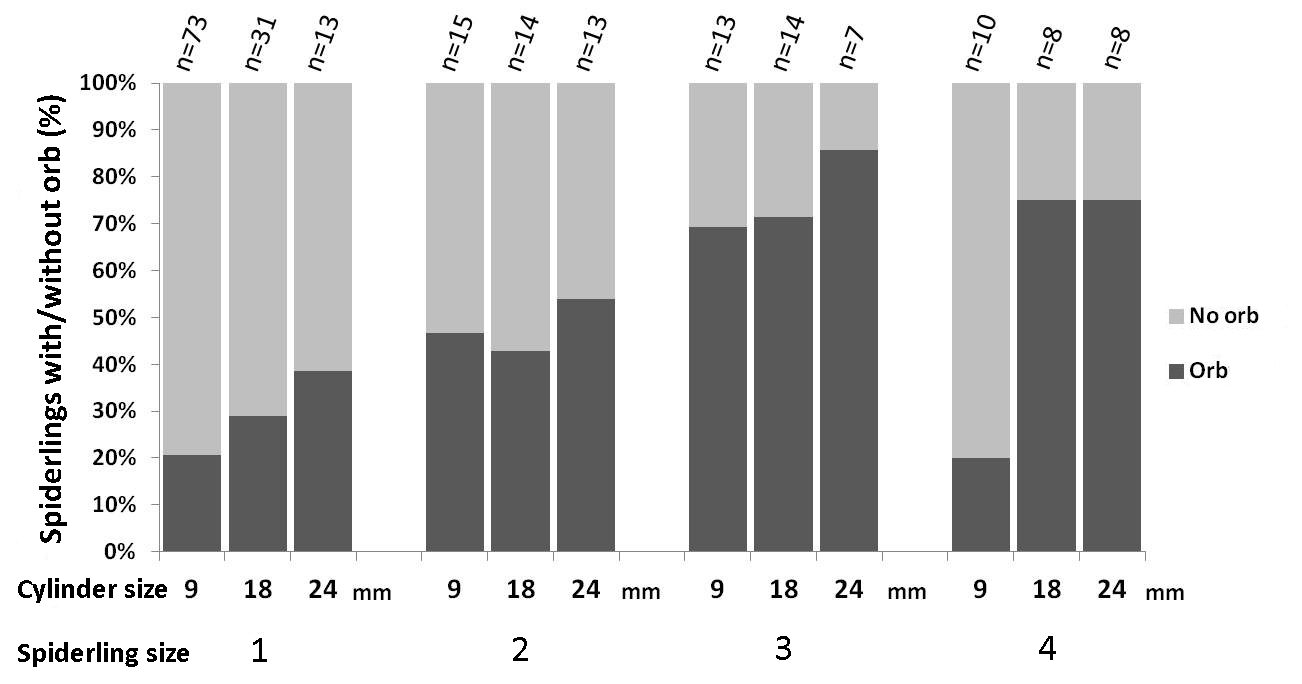
**

**S2 Figure.** Percentage of *Leucauge argyra* spiderlings that built an orb web in 9 mm, 18 mm and 24 mm diameter cylinders (sample sizes are given above each bar).

**Continuation of trends in the field**

In both spiderlings and adults nearly all of the relationships between total web area and different web variables in all webs built in constrained spaces in captivity and in the field, were statistically the same as the relationships of these variables with total web area taking into account only webs built in the field (Table S1). This similarity suggests that the adjustments observed in the laboratory resulted from natural selection favoring similar adjustments in the field, and were not artifacts of captive conditions. In combination with the lack of differences between spiderlings and adults, it also suggests that the adjustments made by spiderlings were the result of their using cues similar to those used by adults to guide their adjustments.

**Table S1**. Web variables measured in orb webs built by *Leucauge argyra* spiderlings and adults in constrained spaces in the laboratory and the field, classified according to whether the adjustments made in the laboratory and in the field represented continuations of adjustments to smaller spaces made in the field. Data on adults from Barrantes & Eberhard [16]. The criterion used to define a continuation was lack of a significant difference between the slope in “allwebs” (webs built both in the laboratory and the field) by a given spider stage category (spiderling or adult) compared with the slope of only field webs of that category (indicated by “✓”). Statistically significant differences between all webs and field webs, implying that adjustments in the laboratory were not continuations of adjustments made in the field, are indicted by “X”.

|  | **Continuation of the adjustment** | |
| --- | --- | --- |
| **Variable** | **Spiderlings** | **Adults** |
| Capture area (mm^2^) | ✓ | ✓ |
| Free zone (mm^2^) | ✓ | ✓ |
| Hub area (mm^2^) | ✓ | ✓ |
| Number of radii | ✓ | ✓ |
| No. hub loops | ✓ | ✓ |
| Web symmetry | ✓ | ✓ |
| Number of frames | ✓ | ✓ |
| No. radii attached to substrate/total radii | X | ✓ |
| Mean radii/frame | ✓ | ✓ |
| No. frame w. single radius/total frames | ✓ | ✓ |
| Capture area/total area | ✓ | ✓ |
| Free zone/total area | X | ✓ |
| Hub area/total area | ✓ | ✓ |
| Mean No. sticky spiral loops | ✓ | Not measured |
| No. sticky spiral loops L+O | ✓ | ✓ |
| Mean sticky spiral space (mm) | ✓ | Not measured |
| Sticky spiral space L (mm) | ✓ | ✓ |
| Mean consistency (mm) | ✓ | Not measured |
| Consistency L (mm) | ✓ | ✓ |
| Frames with spirals beyond frames/total frames | ✓ | Not measured |
